# Personal Value Preferences, Threat-Benefit Appraisal of Immigrants and Levels of Social Contact: Looking Through the Lens of the Stereotype Content Model

**DOI:** 10.3389/fpsyg.2021.609219

**Published:** 2021-03-04

**Authors:** Sophie D. Walsh, Eugene Tartakovsky

**Affiliations:** ^1^Department of Criminology, Bar-Ilan University, Ramat Gan, Israel; ^2^Bob Shapell School of Social Work, Tel Aviv University, Tel Aviv, Israel

**Keywords:** personal values, threat-benefit model, social contact, stereotype content model, asylum seekers and immigrants, Israel

## Abstract

The study examines a model proposing relationships between personal values, positive (i.e., benefits) and negative (i.e., threats) appraisal of immigrants, and social contact. Based on a values-attitudes-behavior paradigm, the study extends previous work on personal values and attitudes to immigrants by examining not only negative but also positive appraisal and their connection with social contact with immigrants. Using a representative sample of 1,600 adults in the majority population in Israel, results showed that higher preference for anxiety-avoidance values (self-enhancement and conservation) was related to higher levels of perceived threat and lower levels of benefit, while higher preference for anxiety-free values (self-transcendence and openness to change) was related to higher levels of perceived benefits and lower levels of threat. Greater opportunities for contact and perceived benefits and lower levels of threats were related to more social contact. The model showed good fit across the total sample, and across four diverse immigrant groups in Israel (diaspora immigrants from the Former Soviet Union, Ethiopia and Western countries, and asylum seekers). In line with a Stereotype Content Model, which suggests that group-specific stereotypes are related to social structural characteristics of the group, associations between variables differed by group. Results strengthen a theoretical conceptualization that posits an indirect relationship between personal value preferences and behavior through group appraisal. They highlight the importance of comprehensive conceptualizations including both positive and negative appraisal of immigrants, which take into account the way different groups may be appraised by the majority population.

## Introduction

Based on an attitude-behavior paradigm (Ajzen and Fishbein, 1977, 1980), theory and empirical research in the area of intercultural relations has focused on the way the attitudes (i.e., cognitions) that we hold toward individuals from a different immigrant, ethnic or racial group can predict how we behave toward and interact with members of those groups. These cognitions can include the stereotypes that we hold (Cuddy et al., 2007) and/or the degree of threat (Stephan and Stephan, 1996, 2000; Stephan et al., 2005) that we perceive these groups to manifest for us. Theories in the area of social psychology have focused on structural characteristics of groups which inform the cognitions we hold, such as their levels of status and perceived competition (in the Stereotype Content Model, SCM (Lee and Fiske, 2006; Fiske et al., 2007; Cuddy et al., 2009) or their realistic or symbolic threat (in the case of Integrative Threat theory, ITT (Stephan and Stephan, 1996, 2000). In addition, extensive research has shown the way in which the personal values that an individual holds can predict the positive or negative attitudes toward immigrants that s/he will hold (Davidov et al., 2008, 2020; Davidov and Meuleman, 2012; Beierlein et al., 2016). The current paper aims to extend previous research by combining these bodies of literature to examine the way in which the relationship between personal values and attitudes may be useful in predicting levels of contact that an individual will choose to have with immigrants in his or her society. Based on a value-attitude-behavior paradigm (Homer and Kahle, 1988), the current study examines a theoretical Threat-Benefit model (Tartakovsky and Walsh, 2016a,b, 2019) in which the Personal Values (Schwartz, 2006; Schwartz et al., 2012) which an individual holds will predict both directly and indirectly (through group appraisal) (Schwartz et al., 2010; Lönnqvist et al., 2013; Roccas and Sagiv, 2017) levels of chosen social contact with members of an immigrant group.

The study examines appraisal of, and contact with, four diverse immigrant groups in Israel, which provides a unique immigration context due to the presence of both formally welcomed or “valued” (Montreuil and Bourhis, 2001) diaspora immigrants from the Former Soviet Union, Ethiopia, and Western countries as well as asylum seekers, mainly from Sudan and Eritrea (Horenczyk and Ben-Shalom, 2006) who have a far less accepted place in Israeli society (Kritzman-Amir, 2009). Previous research has shown that attitudes of the public toward immigrant groups vary according to identity and structural characteristics of the group and according to the context in which the immigration is framed (Hellwig and Sinno, 2017; Gorodzeisky and Semyonov, 2019; Jedinger and Eisentraut, 2020) suggesting the imperative to examine to what extent the proposed model holds across immigrant groups. Specifically, research within the framework of SCM has shown that, in the eyes of the Jewish majority population, these groups show different positions on the axis of competition (i.e., warmth) and status (i.e., competence) (Durante et al., 2013). As such, we also examine the extent to which a values-threat/benefit-contact model differs for groups that vary in their meaning for the host population. In a current reality of large-scale migration and increasingly heterogeneous societies, a theoretically based understanding of what will determine the host population’s willingness to interact and be in contact with immigrants is of critical importance.

### A Threat-Benefit Model for Understanding Appraisal of Immigrants

One of the most popular theories of appraisal of immigrants is the Integrative Threat Theory (ITT) (Stephan et al., 1999; Stephan and Stephan, 2000). The main assumption of the ITT is that local people perceive immigrants as a threat (Stephan and Stephan, 2000). The theory delineates four types of threats that immigrants may represent for local people: realistic threat (competition for resources), symbolic threat (resulting from incompatibility in cultural values), inter-group anxiety (out-group fear), and negative stereotypes (leading to anticipated negative behavior). However, despite its popularity among researchers, ITT can be seen to have two main drawbacks. The main weakness of the theory is in its focus on the exclusively negative aspects of the perception of immigrants. Indeed, empirical studies conducted in different countries have indicated that anti-immigrant attitudes are strong in the local populations (Raijman and Semyonov, 2004; Davidov et al., 2008; Gorodzeisky and Semyonov, 2009). However, they have also demonstrated that most people in the receiving countries have some positive attitudes toward immigrants, and a substantial part of the local population supports immigration to their country (Lee and Fiske, 2006; Mayda, 2006; Leong, 2008; Velasco Gonzalez et al., 2008). As such, ITT relates to attitudes toward immigrants as representing one factor. A one-factor perspective has also been used in empirical studies regarding immigrants (Raijman et al., 2008). However, Threat-Benefit theory (TBT) theorizes (and our previous empirical studies have supported; Tartakovsky and Walsh, 2016a,b) that the levels of threats and benefits that a group represents may be considered as two factors, e.g., diaspora immigrants from the Former Soviet Union in Israel may simultaneously be considered to benefit the country by strengthening social cohesion yet may also represent an economic threat as they may compete over resources, such as jobs and housing. A two-factor model was also found in recent research in Greece in which perceived threat and perceived contribution of asylum seekers predicted attitudes toward permanent settlement (Thravalou et al., 2020). A two-factor perspective leads to the theoretical question of whether it is the perception of threat or, more so, the appreciation of benefits (i.e., positive appraisal) that will predict levels of social contact that an individual will choose to have with members of a particular immigrant group.

Another important limitation of ITT relates to the fact that it does not delineate the antecedent factors for different threats. What is it about the *individual* that leads him or her to perceive a particular immigrant group as representing a threat and/or potential benefit to the receiving society? As will be discussed later, in extension of previous literature (Davidov et al., 2008, 2020; Davidov and Meuleman, 2012; Beierlein et al., 2016), TBT assumes that it is general motivational goals expressed in personal value preferences (Schwartz et al., 2012) which affect the individual’s appraisal of immigrants as threatening or/and beneficial for the receiving society.

The threat-benefit theory (Tartakovsky and Walsh, 2016a,b) delineates four types of threat (economic, physical, social cohesion, modernity) and four benefits (economic, social cohesion, humanitarian, and cultural diversity). *Economic threats* reflect a fear held by local people of losing their dominance over economic resources by competing for jobs, welfare, and other valuable social resources with the immigrants. *Physical threats* reflect a fear held by local people that immigrants may cause physical harm, including harm to the local people’s bodies and property. *Threats to societal cohesion* reflect a fear that immigrants will alter the existing local value system and introduce new behavioral norms, customs, and rituals. *Threats to modernity* reflect the fear that immigrants will bring non-modern values and behavioral norms. *Economic benefits* reflect the immigrants’ potential to contribute to the economic development of the receiving country, which is related to the immigrants’ readiness to take jobs that local people do not want or lack the skills to do, as well as the immigrants’ readiness to work longer hours and for a lower salary. In addition, immigrants may bring valuable skills, language knowledge, and international connections that may benefit the local economy. *Cultural diversity benefits* are related to the new cultural elements (food, clothes, music, etc.) that immigrants bring with them, which may be perceived by some local people as culturally enriching the receiving society. *Humanitarian benefits* are related to the satisfaction of helping immigrants escape danger in their home countries and improve their quality of living. *Social cohesion benefits* are related to the potential ability of some culturally close groups of immigrants (e.g., diaspora Jews coming to Israel) to strengthen the dominant group in the receiving country.

### Personal Values and Behavior: Direct and Indirect Paths

The relationship between personal beliefs and attitudes toward immigrants has been well-studied. In particular, research on the relationship between ideological beliefs and attitudes to immigrants has emphasized the negative association between Right-Wing Authoritarianism (RWA) and Social Dominance Orientation (SDO) on attitudes toward immigrants (Araújo et al., 2020) and the positive association with multi-cultural ideology (MCI) (Grigoryev et al., 2019a). RWA and SDO have been considered to be principle drivers of prejudice and out-group hostility, the first postulating dangerous world beliefs and the second proposing a perspective of the social world as a dangerous jungle (Duckitt and Sibley, 2009; Sinn, 2019) while MCI has been considered to forward universalizing socio-functional motives (Grigoryev et al., 2019a). Recent work emphasizes an interaction between personality variables such as RWA and SDO and context (e.g., country-level threat) (Araújo et al., 2020). The present study draws from Schwartz’s theory of values (Schwartz, 1992, 2006; Schwartz et al., 2012), which defines values as desirable trans-situational goals that serve as guiding principles in people’s lives. Schwartz’s value theory has been suggested to be the best available nomological framework through which to examine ideological beliefs and ideological differences (Sinn, 2019) due to its richness and ability to focus on a wider range of personal beliefs than RWA and SDO.

Value preferences reflect the individual’s general motivational goals, which affect the individuals’ perception of reality and direct behavior (Schwartz, 2006). The theory (Schwartz et al., 2012) specifies a comprehensive set of nineteen motivationally distinct values: power (dominance and resources), achievement, hedonism, stimulation, self-direction (thought and action), universalism (nature, concern, and tolerance), benevolence (caring and dependability), humility, conformity (rules and interpersonal), tradition, security (personal and societal), and face. The theory assumes the existence of dynamic relations between values: the pursuit of each value has consequences that may conflict or may be congruent with the pursuit of other values. The conflicts and congruities among all 12 values yield an integrated structure of four higher-order value types arrayed along two orthogonal dimensions: Openness to change values (including self-direction and stimulation) emphasize readiness for new ideas, actions, and experiences. They contrast with conservation values (including conformity, tradition, and security) that emphasize self-restriction, order, and preserving the status quo. Self-enhancement values (including values of power and achievement) emphasize pursuing one’s interests. They contrast with self-transcendence values (including universalism and benevolence) that emphasize transcending one’s interests for the sake of others. Finally, the theory assumes that the self-transcendence and openness to change values express the goals of growth and self-expansion and are more likely to motivate people when they are free of anxiety. The self-enhancement and conservation values are directed toward protecting the self-against anxiety and threat.

As is assumed in social psychology, attitudes can assert value preferences (Hitlin, 2003; Schwartz, 2006). As such, researchers have assumed that attitudes toward minorities assert some personal values and contradict others (Sagiv and Schwartz, 1995; Schwartz, 2006). Specifically, researchers have argued that self-enhancement values (especially power) may be associated with negative attitudes toward minority groups. People who value self-enhancement tend to perceive the world as a win-lose game (Schwartz, 2006). They sense that they should compete with others and control more people and resources to succeed in life. If others are perceived as threatening to one’s society, it justifies one’s urgency to fight them. Perceiving others as beneficial to society contradicts one’s readiness to take valuable resources from them to advance oneself. On the other hand, self-transcendence values (especially universalism) may be associated with positive attitudes toward these groups, due in part to their relationship with higher levels of empathy (Zibenberg and Kupermintz, 2016). People, for whom helping others is important, prefer to see others as more beneficial and less threatening to their society, because helping somebody who is threatening may cause cognitive dissonance. In addition, values of security and tradition may be associated with negative attitudes toward minority groups, as a need for conservation prevents the individual from perceiving a newcomer, who may shake the status quo, as positive. Lastly, openness to change values may be associated with positive attitudes toward outgroups (Schwartz, 2007), as the change and new elements that the immigrants bring may be seen as positive.

These theoretical assumptions have been mainly supported in empirical studies on attitudes toward immigrants conducted on both the individual level (Feather and McKee, 2008; Leong, 2008; Vecchione et al., 2012), and at the level of societal value preferences (Leong and Ward, 2006; Davidov et al., 2008). Analysis of data from diverse countries in the European Social Survey (ESS) (Jowell et al., 2007) showed that respondents high on conservation values reported more negative attitudes toward immigrants (e.g., did not want them to come to their country) while those high on self-transcendence values reported more positive attitudes toward immigrants (Davidov et al., 2008; Davidov and Meuleman, 2012). However, these relationships have been found to be weaker in countries with high levels of cultural-embeddedness (Davidov et al., 2014). Similarly, individuals high on universalism values and low on group security values in Italy, Germany, and Spain were found to have more positive perceptions of immigrants (e.g., making the country a better place to live) (Vecchione et al., 2012). In addition, a recent analysis of the 2014–2015 ESS suggests that the relationship between values and attitudes toward immigrants may be partially mediated by perceived symbolic threat (Davidov et al., 2020). The current study extends this research to examine the relationships between personal values and benefits (and not just threats) among diverse groups of immigrants and to see how the value-attitude relationship further predicts levels of social contact.

A theoretical overlap between theories of intergroup relations, such as ITT (Stephan and Stephan, 2000), and personal values theory (Schwartz, 2006) can be found in their attention to levels of anxiety and threat, as experienced by the individual. For ITT (Stephan and Stephan, 2000), one of the four elements of threat relates to inter-group anxiety, as aroused by the presence of the immigrant group. In personal values theory, Schwartz (2006) differentiates between anxiety-free values (openness to change and self-transcendence) which relate to self-growth, as opposed to anxiety-avoidance values (self-enhancement and conservation) in which the individual is motivated toward self-protection against anxiety. Schwartz (2010) suggests that if people are preoccupied with pursuing specific values to control their anxiety, they have fewer psychic resources to be open to the “other,” suggesting a relationship between anxiety-avoidance values and negative appraisal of immigrants. In the current study, based on a Threat-Benefit Theory, we suggest that negative (threat) appraisal of immigrants can allow attainment, expression, or fulfillment of anxiety avoidance values while positive (benefit) appraisal can enable the attainment or fulfillment of anxiety-free values.

Recent years have seen a development of theory and research on the relationship between personal values and behaviors (Bardi and Schwartz, 2003; Roccas and Sagiv, 2010). Empirical studies have shown values as related to diverse behaviors, such as voting patterns (Schwartz et al., 2010), helping behavior (Daniel et al., 2014), prosocial behaviors (Lönnqvist et al., 2013), political activism (Vecchione et al., 2015), and adolescent aggression (Benish-Weisman, 2015). Yet the mechanisms behind the relationship have been understudied. In their recent book, Roccas and Sagiv (2017) suggest that the relationship between values and behavior can be both direct and indirect, mediated by attitudes (Grunert and Juhl, 1995), valences (Feather and McKee, 2008), and perception or interpretation (Schwartz et al., 2000). Yet although limited research has examined the relationship between values and intentions for social contact (Sagiv and Schwartz, 1995), no research to date has explored an indirect path of appraisal of groups in the relationship between values and social contact. In the current study, we examine direct and indirect (through threat/benefit appraisal of immigrants) paths in the relationship between personal values and social contact with the immigrants.

### Immigrants and Asylum Seekers in Israel Through the Lens of a Stereotype Content Model

In the current study, we examine the proposed model across four diverse immigrant groups. Research in Britain (Hellwig and Sinno, 2017) and Germany (Jedinger and Eisentraut, 2020) suggests that the threats that members of the majority group may feel toward different immigrant or minority groups differ, in line with perceived characteristics of the immigrant group (e.g., cultural, religious, economic). In addition, recent research in Germany (Landmann et al., 2019) also suggests that there may be particular threats that relate to refugee groups (e.g., safety, cohesion, prejudice, and altruistic threats) over and above the more traditionally conceived symbolic and realistic threats (Stephan et al., 2000). Moreover, research also suggests that these different threat perceptions may lead to different behaviors (De Rooij et al., 2018), making it important to examine how the particular relationships between values, immigrant appraisal, and social contact may differ across groups.

Recent years have seen the development of theory and empirical research around the Stereotype Content Model (SCM) (Lee and Fiske, 2006; Fiske et al., 2007; Cuddy et al., 2009). Broadly speaking SCM suggests that the stereotypes held toward particular immigrant groups are not unidimensional (good/bad) (Allport, 1954) but rather are defined along axes related to social structural characteristics of the group in question (Cuddy et al., 2009). In particular, the nature or content of the stereotype will be related to the extent to which we feel a group intends to harm us and the extent to which we feel they are capable of harming us. These two questions lead to two axes: warmth (e.g., how friendly, good-natured, sincere, and warm we perceive the group to be) which is related to the potential harm or benefit which we assess the group as representing, and competence (e.g., how capable, confident, skillful we assess the group to be) which relates to whether we believe the target group members can effectively enact the threat (Fiske et al., 1999; Fiske, 2018). Higher social status groups are generally perceived as more competent while more competitive groups are perceived as lower in warmth (Oldmeadow and Fiske, 2010). In addition, in countries with high-income inequality (such as Israel), low socio-economic status has been related to low competence but high warmth (Durante et al., 2017). Interestingly, Sevilleno and Fiske (2012) suggest that the dimensions of warmth and competence approximately parallel the underlying motivations behind RWA and SDO.

SCM stems from a basic premise that outgroups are not a monolithic body, but rather differ among themselves and represent differing threats to the population, according to perceived or actual economic, geographic, power and race relations and particular threats (Gorodzeisky and Semyonov, 2019). Stereotypes can be cultural or consensual (i.e., shared by members of a particular culture) or personal (i.e., represent the individual’s views about a particular group) (Findor et al., 2020). Cultural stereotypes, in particular, those based on demographics such as race or ethnicity have been found to be more accurate than personal stereotypes (Jussim et al., 2015), and are likely to be more connected with real positions of groups in the social structure. Personal stereotypes also tend to be more positive as they are not affected by factors such as social desirability (Kotzur et al., 2020). From a functional perspective (Neuberg et al., 2020), stereotypes play a role in enabling members of a society to identify and manage social and physical opportunities and threats which a particular group may manifest and thereby influence behavior. Stereotypes allow members of a society to assess to what extent a particular group can enable or hinder the goals. In line with SCM which focuses on cultural stereotypes (Findor et al., 2020) and a functional perspective (Neuberg et al., 2020), we assume that threat-benefit appraisal is related to structural characteristics of a group that may hinder or enable opportunities or threats for the host society (i.e., will differ by group), and which parallel underlying personal motivations (Sevillano and Fiske, 2012). As such, the extent to which threat-benefit appraisal may be salient (i.e., explain the relationship between values and contact) to the local population may vary by immigrant group and their particular perceived social structural characteristics.

Israel is a unique context in which to examine attitudes to immigrants, due to the combination of diaspora or supposedly desired immigrants (Titzmann and Stoessel, 2014), as well as more negatively viewed labor migrants and asylum seekers (Kritzman-Amir, 2012). A more nuanced examination shows varying attitudes toward the different groups, related to desirability, size, perceived resources, and possibilities for contact. In the current study, we examine appraisal of four groups of immigrants: three diaspora immigrant groups—from the FSU, Ethiopia, and Western countries such as France, the United Kingdom, and the United States—and a fourth group of asylum seekers who can be seen as occupying diverse positions along warmth-competence axes (Fiske et al., 2007). These four groups are the largest groups that have immigrated to Israel in recent years, and recent research shows they differ significantly in the levels of threat/benefit that the host population perceives them as representing (Tartakovsky and Walsh, 2019).

Together with their children born in Israel, immigrants from the FSU number about 1,100,000 Central Bureau of Statistics (2020)^1^ ^,2^, while immigrants from Ethiopia number about 137,000. Since 1989 there have been approximately 66,000, 69,000, and 23,000 immigrants from France, the United States, and the United Kingdom, respectively Central Bureau of Statistics (2020)^2^, and asylum seekers number about 55,000 (UNHCR, 2013). These groups differ drastically in their socio-demographic characteristics, the support they receive from the government, and their social and psychological adjustment in Israel (Afeef, 2009).

The four groups also differ in how they are perceived by the Israeli majority population. The three diaspora groups do, at least formally, enjoy a desired status among the Israeli majority population (Avineri et al., 2009; Titzmann and Stoessel, 2014). Recent research (Tartakovsky and Walsh, 2019) revealed that while the appraisal of asylum seekers in Israel is characterized by multi-domain negativity, attitudes toward diaspora groups are more nuanced and relate to the particular characteristics of the immigrant group. Immigrants from Western countries are most favorably appraised for their high levels of Jewish identity, patriotism, and social resources which they bring (Amit, 2012); appraisal of immigrants from the FSU can be described by ambivalence, as they are seen as bringing high levels of both threats (cultural, political, economic) but also benefits (economic, social cohesion) (Tartakovsky and Walsh, 2019). Immigrants from Ethiopia are appraised both by bringing some benefits (humanitarian, social cohesion) but also perceived negatively by some of the local population for their limited resources and higher levels of crime (Kahan-Strawczynski et al., 2013).

Many immigrants from FSU came with high levels of education and human capital, and studies have documented impressive levels of employment and integration (Amit, 2012; Remennick, 2012). Yet they have been subject to discrimination based on their perceived symbolic and realistic threat (Tartakovsky and Walsh, 2016a) and questioned Jewish status (Remennick, 2012). The waves of immigration from Ethiopia since the 1980s have been characterized by difficulties in integration resulting from deep cultural differences (Kaniel, 1990; Tannenbaum, 2008), as well as racism and discrimination based on skin color (Offer, 2007). On the other hand, the Ethiopian Jewish community came to Israel with a strong Jewish identity and rich culture and heritage (Schwarz, 2016). Large waves of immigrants from France have brought many religious, ideologically oriented immigrants (Ben-Rafael and Schmid, 2007), many of whom maintain a transnational identity (Amit, 2012). Little has been written about English-speaking immigrants to Israel (Walsh and Horenczyk, 2001), whose immigration is also largely driven by religious and Zionist motivations and who bring with them high levels of human capital. In contrast, while immigrants from the FSU, Ethiopia, and Western countries receive economic and psychosocial support from the state, asylum seekers have limited rights in Israel and receive no help from the state in their adjustment (Kritzman-Amir, 2009). The majority of the asylum seekers have come from Eritrea and Sudan, following significant experiences of trauma (Nakash et al., 2015), though their entrance to Israel has been highly controversial, with many parts of the society calling for their deportation.

Research within the framework of SCM (Durante et al., 2013) has suggested that FSU immigrants represent relatively high competence (e.g., high human capital) and low warmth (high economic and social cohesion threats), Ethiopian immigrants represent low competence (low status) but high warmth (little competition), Western immigrants represent high competence (high status) and high warmth (e.g., shared social and religious ideals) asylum seekers represent low competence (low status) and low warmth (high threats to personal safety and the Jewish character of Israel). One of the questions of the current study is to what extent, in the light of these differences, will a threat-benefit model differ across groups. SCM suggests that in cases in which there is little actual contact between the groups, stereotypes will play a greater role in the evaluation of other groups (Grigoryev et al., 2019b). In the case of Israel, due to the smaller numbers and geographical concentration, local population members have less actual contact with asylum seekers and Ethiopian immigrants.

### The Current Study

**FIGURE 1:**
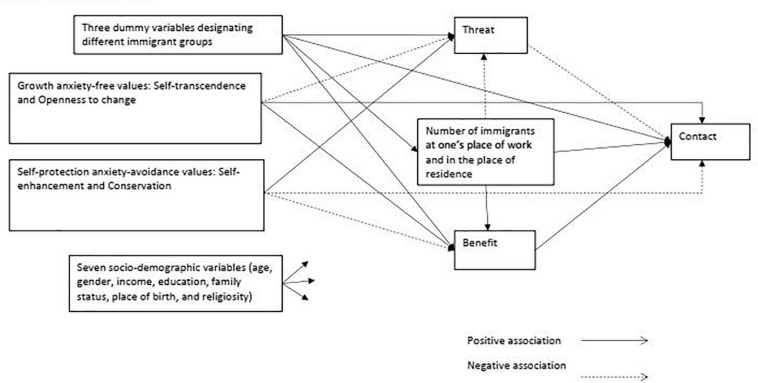
The research model.

The current study examines a theoretical model focusing on dispositional characteristics of the individual (personal values) as predicting levels of social contact directly and indirectly through the association with the positive and negative appraisal of immigrant groups (see Figure 1). In line with theory and the results of previous studies, we hypothesized that:

(1) Higher levels of growth anxiety-free values (openness to change and self-transcendence) would directly predict higher levels of perceived benefits, lower levels of perceived threats, and higher levels of social contact.

(2) Higher levels of self-protection anxiety-avoidance values (conservation and self-enhancement) would directly predict higher levels of perceived threat, lower levels of perceived benefit, and lower levels of social contact.

(3) Perceived benefit would positively predict social contact while perceived threat would negatively predict social contact.

(4) Personal values would indirectly predict the level of social contact through threats and benefits. As such, higher levels of perceived benefits and lower levels of threats would partly explain the positive relationship between anxiety-free values and social contact, while higher levels of perceived threat and lower levels of perceived benefits would partly explain the negative relationship between anxiety-avoidance values and social contact.

In addition, as suggested above, following the testing of the overall theoretical model, based on ideas from SCM, we also explored the extent to which the proposed model varied across diverse groups.

## Methods

### Participants and Procedures

The study involved a representative sample of 1,600 adults ranging from 18 to 91 years of age (Mean age = 44.2 years, *SD* = 17.0; 51.7% female) of the majority Jewish population in Israel^3^. Of the participants 26% were single, 59% married, 9.7% divorced and 4.6% widowed. Eighty-five percent had graduated high school with a high school certificate and 35% had an academic degree. In terms of religious identification, 50% reported being secular or atheist, 31% traditional and 19% religious. Sixty-eight percent were born in Israel, 15.8% in the FSU, and 1% in Ethiopia. The remaining 15% were born outside of Israel in other countries. Sampling involved random route sampling (De Rada and Martín, 2014) across the whole of Israel inside the internationally recognized borders. The response rate was 69%, akin to those of similar surveys such as the European Social Survey (De Rada and Martín, 2014). A comparison with the Central Bureau of Statistics data regarding the Israeli Jewish population Central Bureau of Statistics (2020)^2^ confirmed the sample’s representativeness.

Completion of questionnaires was face to face in the participant’s home with a research assistant who returned up to three times to each sampled house. The questionnaires were in Hebrew; however, Russian-, Amharic-, and English-speaking interviewers were available to help those participants who had difficulties with Hebrew. Four versions were distributed. Each version asked the participants as to their answers toward one of four immigrant groups: Diaspora immigrants from the Former Soviet Union, Ethiopia, and Western countries (such as France, the United Kingdom, and the United States) and asylum seekers. The choice between the four groups in the study was randomized; however, those participants who were immigrants or children of immigrants did not answer the questionnaire regarding their own group. Thus, 400 questionnaires were received for each of the four immigrant groups in the study: Immigrants from the FSU, Ethiopia, Western Countries, and asylum seekers from African countries. Ethical approval was gained from the IRB of Bar Ilan University. Participation was voluntary and participants did not receive compensation for survey completion. Data was collected by the PORI research company^4^ and the survey was funded by a grant from the Israel Science Foundation.

### Measures

#### Personal Value Preferences

The personal value preferences were measured using the latest version of the Portrait Values Questionnaire, PVQ-R (Schwartz et al., 2012). This questionnaire consists of 57 items. Each item portrays a person’s goals, aspirations, or wishes that indicate the importance of a specific value. For each item, respondents indicate how similar the described person is to them on a 6-point scale, from 1 (*not like me at all*) to 6 (*very much like me*). Item example: “It is important to him to avoid upsetting other people” (conformity). The questionnaire was tested in ten countries including Israel and has demonstrated good psychometric properties (Schwartz et al., 2012). Reliabilities for all value scales in the current study were satisfactory (as measured by Cronbach’s alpha): openness to change (α = 0.84), self-transcendence (α = 0.89), conservation (α = 0.86), and self-enhancement (α = 0.84), for the pooled sample.

#### Immigrants’ Appraisal

Appraisal of immigrants was measured by the Threats-Benefits Inventory (TBI, see Tartakovsky and Walsh, 2016a,b, 2019 for details of the development of the inventory). The questionnaire consists of 35 items which are measured on a 5-point scale, from 1 (*strongly disagree*) to 5 (*strongly agree*)^5^. Threats were examined in four areas: Economic threats (e.g., “Immigrants drain welfare funds”); Physical threats (e.g., “Immigrants commit many violent crimes against Israelis”); Threats to social cohesion (e.g., “Immigrants are a threat to the Jewish character of Israel”); Threats to modernity (e.g., “Immigrants bring non-progressive rules of raising children, e.g., physical punishment”). ***Benefits*** are measured in four areas: Economic benefits (e.g., “Immigrants have a stronger work motivation than Israelis”), Cultural diversity benefits (e.g., “Immigrants bring cultural diversity to our population and allow us to learn about cultures we might never learn about otherwise”); Humanitarian benefits (e.g., “Accepting immigrants can help to save lives”), and Social cohesion benefits (e.g., “Immigrants strengthen the Jewish character of Israel”). The structural validity of the questionnaire and its measurement invariance across different immigrant groups in Israel was confirmed in two previous studies conducted among social workers (Tartakovsky and Walsh, 2016a,b), as well as in the general sample (Tartakovsky and Walsh, 2019). In the current study, the two higher-order indexes of perceived general threat and benefit were included in the tested theoretical model. Alpha Cronbach for benefits and threats were as follows: Benefits: entire sample −0.91, Asylum seekers −0.88, Ethiopians −0.89, Western counties −0.86, FSU −0.86. Threats: entire sample −0.93, Asylum seekers −0.91, Ethiopians −0.91, Western countries −0.92, FSU −0.90.

#### Opportunities for Contact

Opportunities for contact with immigrants were measured by two questions: “How many immigrants are there in your workplace”?; “How many immigrants are there in your neighborhood”? [1- none (0%); 5—a great number (30% or more)]. The correlations between the two opportunity for contact variables were low (between .031 and −0.43 for the four groups), so they remained as separate variables.

#### Social Contact

Social contact was measured by a scale developed based on contact with immigrant questionnaires by Islam and Hewstone (1993), Voci and Hewstone (2003), and (Walsh et al., 2018). Some of the items were also taken from the Contact with Disabled Persons Scale (CDP) (Yuker and Hurley, 1987). The participants were asked to report how frequently they were in social contact with immigrants during the last year. The questionnaires contained 12 items measured on a 5-point scale from 1—*never* to 5—*very often*. Items included “You socialized at the home of an immigrant,” “You shared something personal with an immigrant.” Cronbach’s alpha for the social contact was 0.95 for the total sample ranging from 0.86 for asylum seekers to 0.94 for FSU immigrants.

### Data Analysis

Data analysis was conducted using Structural Equation Modeling (SEM; AMOS 22, Arbuckle, 2014). In the analysis, we used observed variables due to the high internal consistency of the variables in the study and a large number of variables in the model. The covariance structure of the hypothesized model was evaluated with multiple fit indexes, and the following values were regarded as indicating a good fit: CFI > 0.90, and RMSEA < 0.08 (Hooper et al., 2008; Brown, 2014). The direct, indirect, and total effects were tested using the bootstrapping method with 1,000 re-samples (Arbuckle, 2014). Due to a large number of connections in the model and a large research sample, we demanded *p* < 0.01 as a minimal level of significance (Hochberg and Benjamini, 1990). The number of missing values in the sample was small (less than 2% for each variable), and missing data were handled using Maximum Likelihood Estimation (Allison, 2012; Arbuckle, 2014). Following SEM using the pooled sample, multi-group SEM was used to see in what way the overall model was similar or differed across the four groups.

## Results

### Descriptive Statistics

Table 1 presents means, standard deviations, and the results of ANOVAs of all the variables in the study compared across the four immigrant groups. The participants reported the following order in the number of immigrants at their work and the residential area (from largest to smallest): immigrants from the FSU, immigrants from western countries, immigrants from Ethiopia, and asylum seekers. The appraisal of immigrants by local people also differed across the immigrant groups: immigrants from western countries were perceived as the least threatening and most beneficial for Israeli society, while asylum seekers were perceived as the most threatening and least beneficial. Finally, the frequency of social contact between locals and the immigrants also varied across immigrant groups: the most frequent social contact was with immigrants from the FSU, followed by immigrants from western countries, Ethiopia, and asylum seekers. Table 2 shows the correlations between the study variables.

**TABLE 1:** Comparison across four immigrant groups of opportunities for contact, threat-benefit appraisal and social contact [Means (SD) and ANOVA].

Variables	Asylum seekers	Ethiopians	Western countries	Former Soviet Union	*F*(3, 1,596); *p;* partial η^2^
Number of immigrants at work	1.47 (1.21)^d^	1.96 (1.26)^c^	2.39 (1.53)^b^	2.73 (1.31)^a^	67.2; 0.000; 0.112
Number of immigrants in the residential area	1.49 (0.61)^d^	2.08 (0.95)^c^	2.39 (0.89)^b^	2.86 (0.92)^a^	182; 0.000; 0.255
Perceived threat	3.00 (0.70)^a^	2.39 (0.62)^c^	2.06 (0.62)^d^	2.50 (0.59)^b^	153; 0.000; 0.223
Perceived benefit	2.38 (0.61)^d^	3.33 (0.63)^c^	3.56 (0.59)^a^	3.45 (0.57)^b^	322; 0.000; 0.377
Frequency of social contacts	1.21 (0.35)^d^	1.68 (0.64)^c^	2.17 (0.80)^b^	2.36 (0.82)^a^	234; 0.000; 0.306

*Different superscript letters mean that there is a statistically significant difference between the groups (*p* < 0.05).*

**TABLE 2:** Correlations between the study variables (pooled sample).

	1	2	3	4	5	6	7	8	9	10	11	12	13	14	15
Age (1)	−														
Gender (2)	0.044	−													
Family status (3)	0.623**	0.084**	−												
Education (4)	0.082**	–0.026	0.067**	−											
Religiosity (5)	–0.013	0.000	0.009	−0.131**	−										
Income (6)	0.167**	−0.204**	0.131**	0.400**	−0.097**	−									
Country of origin (1-Israel; 0-other) (7)	0.419**	0.005	0.291**	0.107**	−0.071**	0.076**	−								
Number of immigrants at work (8)	−0.075**	–0.027	−0.070**	0.061*	−0.057*	0.134**	−0.076**	−							
Number of immigrants in the residential area (9)	–0.001	–0.011	–0.049	–0.001	–0.023	0.018	0.015	0.524**	−						
Openness to change (10)	−0.108**	–0.046	−0.071**	0.022	−0.156**	0.032	−0.111**	0.077**	0.049	−					
Self-enhancement (11)	−0.112**	−0.098**	−0.063*	0.010	−0.083**	0.115**	–0.044	0.062*	–.018	0.553**	−				
Conservation (12)	0.124**	0.031	0.101**	−0.069**	0.265**	0.015	–0.026	0.023	0.053*	0.289**	0.122**	−			
Self -transcendence (13)	0.039	0.057*	0.029	–0.001	–0.046	0.003	−0.080**	0.039	0.065**	0.531**	0.130**	0.686**	−		
Benefit (14)	0.039	0.036	0.003	0.066**	−0.067**	0.069**	−0.063*	0.307**	0.297**	0.159**	0.036	0.065**	0.206**	−	
Threat (15)	0.005	−0.051*	0.009	−0.115**	0.153**	–0.010	0.046	−0.113**	−0.152**	−0.084**	0.141**	0.040	−0.119**	−0.501**	−
Contact (16)	−0.101**	–0.001	−0.105**	0.069**	−0.060*	0.089**	−0.094**	0.594**	0.537**	0.126**	0.029	–0.031	0.066**	0.482**	−0.304**

*p < 0.05; **p < 0.10.

### Testing of the Research Model

The research hypotheses were tested by analyzing the research model (Figure 1) in the entire sample (*n* = 1,600). The research model included the following variables: two growth anxiety-free higher-order values (self-transcendence and openness to change), two self-protection anxiety-avoidance higher-order values (self-enhancement and conservation), two higher-order indexes of the immigrants’ appraisal (threat and benefit), and the variable measuring social contact with immigrants. In addition, seven socio-demographic variables (gender, age, education, income, family status, place of birth, and religiosity) were included in the model as control variables; they were connected to the values, threat, benefit, and contact. For details of the relationships between socio-demographic variables and appraisal and contact in the overall model and for each group see the online Supplementary Appendix.

Finally, three dummy variables reflecting the distribution of the respondents into four groups, each reporting appraisal of and contacts with one of the four immigrant groups, were also included as controls in the model. The dummy variables were connected to threat, benefit, opportunities for contact, and contact. The four values were assumed to be correlated between themselves, as well as threat and benefit, and the number of immigrants at one’s place of work and in the residential area.

**FIGURE 2:**
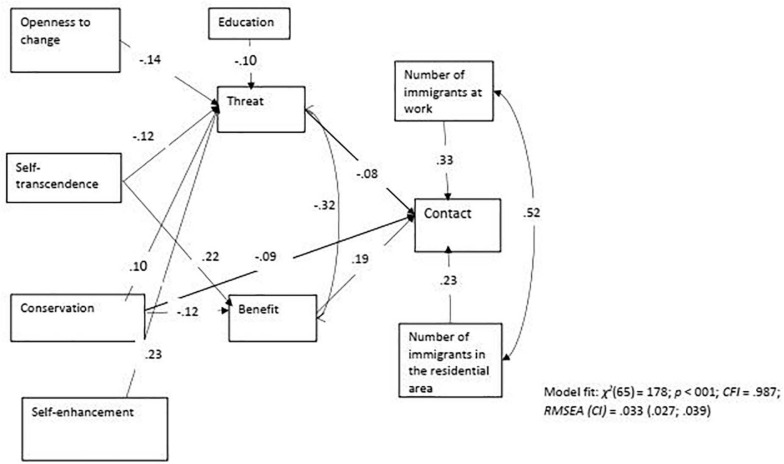
The best fit model with statistically significant direct effects without sociodemographic variables (pooled sample *N* = 1,600). Gender, age, education, income, family, status, place of birth, and religiosity were included as covariates but these paths are not shown for clarity; they are available in Supplementary Figure A1.

The model’s goodness of fit indexes indicated a good fit: χ^2^(65) = 178; *p* < 001; *CFI* = 0.987; *RMSEA (CI)* = 0.033 (0.027; 0.039). The model explained a significant proportion of variance in the three predicted variables (all *p* < 0.01): contact (54%), threat (30%), and benefit (43%). Tables 3–5 present standardized direct, indirect, and total effects of predicting variables on the values, benefit, threat, and contact in the pooled sample. The path diagram in Figure 2 presents the significant direct effects between all variables in the model^6^.

**TABLE 3:** Standardized direct effects: estimate, standard error, and level of significance (pooled sample).

Predicting variables	Contact	Threat	Benefit	Openness to change	Self-transcendence	Conservation	Self-enhancement
Threat	−0.08 (0.02)**						
Benefit	0.19 (0.02)**						
Openness to change	0.06 (0.03)*	−0.14 (0.03)**	0.06 (0.03)				
Self-transcendence	0.02 (0.03)	−0.12 (0.04)**	0.22 (0.03)**				
Conservation	−0.09 (0.03)**	0.10 (0.03)**	−0.12 (0.03)**				
Self-enhancement	−0.02 (0.02)	0.23 (0.03)***	−0.01 (0.02)				
Number of immigrants in the residential area	0.23 (0.02)**	−0.01 (0.03)	0.01 (0.02)				
Number of immigrants at work	0.33 (0.02)***	0.02 (0.03)	0.06 (0.03)*				
Gender	0.01 (0.02)	−0.03 (0.02)	0.03 (0.02)	−0.04 (0.03)	0.05 (0.03)*	0.03 (0.03)	−0.08 (0.03)**
Age	−0.06 (0.02)*	0.00 (0.03)	0.06 (0.02)*	−0.05 (0.03)	0.09 (0.03)**	0.15 (0.03)**	−0.11 (0.03)**
Education	0.03 (0.02)	−0.10 (0.02)**	0.03 (0.02)	0.00 (0.03)	−0.03 (0.03)	−0.03 (0.03)	−0.03 (0.03)
Religiosity	0.03 (0.02)	0.10 (0.02)**	−0.01 (0.02)	−0.15 (0.02)**	−0.05 (0.03)*	0.26 (0.02)**	−0.07 (0.02)**
Income	0.04 (0.02)	0.04 (0.02)	0.05 (0.02)*	0.04 (0.03)	0.01 (0.03)	0.02 (0.03)	0.12 (0.03)**
Marital status	−0.06 (0.02)*	0.02 (0.02)	−0.02 (0.02)	−0.08 (0.03)**	−0.01 (0.03)	0.02 (0.03)	−0.05 (0.03)
Place of birth	0.01 (0.02)	−0.04 (0.03)	0.03 (0.02)	0.10 (0.03)**	0.12 (0.03)***	0.07 (0.03)*	0.01 (0.03)
Group: Asylum seekers	−0.13 (0.03)**	0.55 (0.03)***	−0.65 (0.02)***				
Group: ethiopian	−0.14 (0.02)**	0.18 (0.03)**	−0.15 (0.02)**				
Group: FSU	0.02 (0.02)	0.26 (0.03)***	−0.08 (0.02)**				

*p < 0.05; **p < 0.01; ***p < 0.001. Gender: 1–male; 2–female. Marital status: 0–single; 1–living with a partner. Place of birth: 0–foreign born; 1–born in Israel.

**TABLE 4:** Standardized indirect effects: estimate, standard error, and level of significance (pooled sample).

Predicting variables	Contact	Threat	Benefit
Openness to change	0.02 (0.01)**		
Self-transcendence	0.05 (0.01)**		
Conservation	−0.03 (0.01)**		
Self-enhancement	−0.02 (0.01)**		
Number of immigrants in the residential area	0.01 (0.01)		
Number of immigrants at work	0.01 (0.01)		
Gender	0.01 (0.01)	−0.03 (0.01)*	0.01 (0.01)
Age	0.01 (0.01)	−0.01 (0.01)	0.01 (0.01)
Education	0.02 (0.01)**	−0.01 (0.01)	0.01 (0.01)
Religiosity	−0.05 (0.01)**	0.04 (0.01)**	−0.05 (0.01)**
Income	0.01 (0.01)	0.02 (0.01)**	0.01 (0.01)
Marital status	−0.01 (0.01)*	0.01 (0.01)	−0.01 (0.01)
Place of birth	0.02 (0.01)*	−0.02 (0.01)**	0.02 (0.01)***
Group: Asylum seekers	−0.17 (0.02)**		
Group: Ethiopian	−0.04 (0.01)**		
Group: FSU	−0.04 (0.01)**		

*p < 0.05; **p < 0.01; ***p < 0.001. Gender: 1–male; 2–female. Marital status: 0–single; 1–living with a partner. Place of birth: 0–foreign born; 1–born in Israel.

**TABLE 5:** Standardized total effects: estimate, standard error, and level of significance (pooled sample).

Predicting variables	Contact	Threat	Benefit	Openness to change	Self-transcendence	Conservation	Self-enhancement
Threat	−0.08 (0.02)**						
Benefit	0.19 (0.02)**						
Openness to change	0.08 (0.03)**	−0.14 (0.03)**	0.06 (0.03)				
Self-transcendence	0.07 (0.03)*	−0.12 (0.04)**	0.22 (0.03)***				
Conservation	−0.12 (0.03)**	0.10 (0.03)**	−0.12 (0.03)**				
Self-enhancement	−0.04 (0.02)	0.23 (0.03)**	−0.01 (0.02)				
Number of immigrants in the residential area	0.23 (0.02)***	−0.01 (0.03)	0.01 (0.02)				
Number of immigrants at work	0.34 (0.02)**	0.02 (0.03)	0.06 (0.03)*				
Gender	0.02 (0.02)	−0.04 (0.02)	0.04 (0.02)*	−0.04 (0.03)	0.05 (0.03)*	0.03 (0.03)	−0.08 (0.03)**
Age	−0.06 (0.02)**	−0.01 (0.03)	0.06 (0.02)*	−0.05 (0.03)	0.09 (0.03)**	0.15 (0.03)**	−0.11 (0.03)**
Education	0.05 (0.02)**	−0.11 (0.02)**	0.02 (0.02)	−0.01 (0.03)	−0.03 (0.03)	−0.03 (0.03)	−0.03 (0.03)
Religiosity	−0.02 (0.02)	0.14 (0.02)**	−0.06 (0.02)**	−0.15 (0.02)**	−0.05 (0.03)*	0.26 (0.02)**	−0.07 (0.02)**
Income	0.04 (0.02)	0.06 (0.02)*	0.05 (0.02)*	0.04 (0.03)	0.00 (0.03)	0.02 (0.03)	0.12 (0.03)**
Marital status	−0.07 (0.02)*	0.02 (0.02)	−0.03 (0.02)	−0.08 (0.03)**	−0.01 (0.03)	0.02 (0.03)	−0.05 (0.03)
Place of birth	0.03 (0.02)	−0.05 (0.03)*	0.05 (0.02)*	0.10 (0.03)**	0.12 (0.03)***	0.07 (0.03)*	0.01 (0.03)
Group: Asylum seekers	−0.30 (0.02)**	0.55 (0.03)***	−0.65 (0.02)***				
Group: Ethiopian	−0.18 (0.02)**	0.18 (0.03)**	−0.15 (0.02)**				
Group: FSU	−0.01 (0.02)	0.26 (0.03)***	−0.08 (0.02)**				

*p < 0.05; **p < 0.01; ***p < 0.001. Gender: 1–male; 2–female. Marital status: 0–single; 1–living with a partner. Place of birth: 0–foreign born; 1–born in Israel.

### Predicting Social Contact

We hypothesized that personal value preferences of members of the receiving society would affect their contact with immigrants both directly (Hypotheses 1–2) and indirectly (Hypothesis 4). The results obtained demonstrated that only the direct effect (controlling for opportunities for contact, and socio-demographic variables) of the conservation value on contact was significant. Thus, the hypothesis regarding the direct connection between personal value preferences of members of the receiving society and their contact with immigrants was only partially supported. On the other hand, the indirect effects of all four values on contact (hypothesis 4) were significant (see Table 4). As can be seen in Figure 2, conservation and self-transcendence had indirect effects through both threats and benefits. Conservation values were negatively associated to contact through higher levels of threats (β = −0.008; *p* = 0.003) and lower levels of benefits (β = −0.023; *p* < 0.001). Self-transcendence values were positively associated with contact through lower levels of threats (β = 0.01; *p* = 0.002) and higher levels of benefits (β = 0.042; *p* < 0.001). In both cases, the benefits’ path was stronger than the threats’ path. Openness to change values were positively associated with contact through lower levels of threat, while self-enhancement values were negatively associated with contact through higher levels of threat. Thus, hypothesis 4 was fully confirmed.

We further hypothesized that the appraisal of immigrants as beneficial or threatening for the receiving society would predict social contact with them (Hypothesis 3). The results obtained demonstrated that the direct paths between both components of appraisal to contact were significant: as predicted, the path was positive for benefit and negative for threat. Thus, hypothesis 3 regarding the connection between appraisal and contact was fully confirmed.

### Predicting Threat and Benefit Appraisal

We hypothesized that indirect effects of values on contact would be due to the connection between values and immigrant appraisal (hypothesis 4). The results obtained demonstrated that the direct effects of all values on threat were significant. In addition, the direct effects of two values on benefit were significant: self-transcendence and conservation. Thus, the hypotheses (1–2) regarding the connection between the values of members of the receiving society and their appraisal of immigrants were mostly confirmed.

### Testing for the Similarities and Differences Across Immigrant Groups

We tested similarities and differences in the connections between variables across the four immigrant groups using Multi-Group Structural Equation Modeling (AMOS 22, Arbuckle, 2014). After the goodness of fit of the multi-group model was established, the model’s equivalence across the four groups was tested comparing the constrained model (assuming the equivalence of the path coefficients across the four groups) with the unconstrained model (Selig et al., 2008). Finally, the direct, indirect, and total effects of all the variables in the model on contact with immigrants in each group were tested using the bootstrapping method with 1,000 re-samples with a 95% confidence interval (Arbuckle, 2014).

**FIGURE 3:**
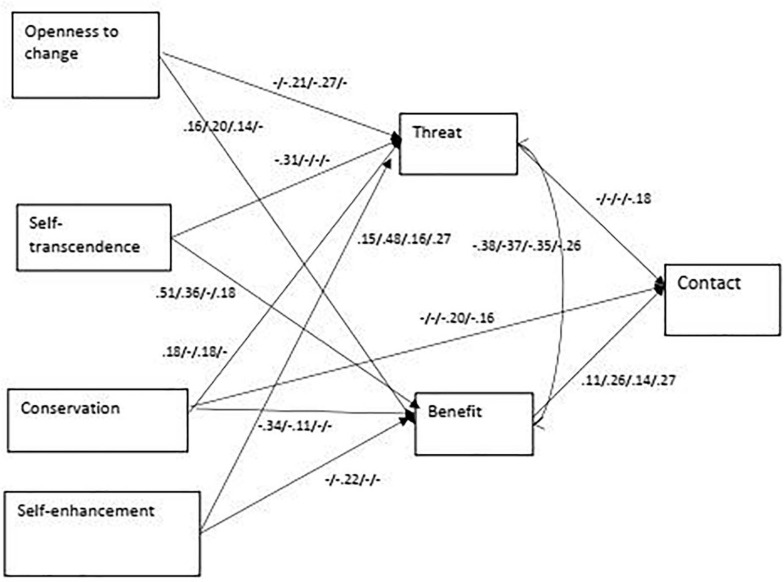
The best fit model of values, threat-benefit apprasial and contact with statistically significant direct effects for the four groups Asylum seekers/Ethiopian/Western countries/FSU (*N* = 1,600).

The multi-group model’s goodness of fit indexes indicated a good fit: χ^2^(83) = 193; *p* < 001; *CFI* = 0.978; *RMSEA(CI)* = 0.029 (0.024; 0.034). The model’s equivalence across the four groups was tested comparing goodness of fit indexes of unconstrained and constrained models (Byrne et al., 1989). The comparison indicated that the constrained model had a significantly worse fit [Δχ^2^(198) = 444, *p* < 0.001]; therefore, the path coefficients were significantly different across the four immigrant groups. Supplementary Tables A1–A3 in the online supplement present standardized direct, indirect, and total effects of predicting variables on the threat, benefit, and social contact for each of the four groups. Figure 3 presents significant direct effects for the four groups.

### Direct Effects of Threat-Benefit Appraisal and Values on Contact

The results obtained demonstrate that the direct effect of threat on contact was significant only regarding immigrants from the FSU (β = −0.176; *p* = 0.003); however, the direct effects of benefit on contact were positive and significant for all diaspora immigrant groups: immigrants from Ethiopia (β = 0.263; *p* = 0.004), western countries (β = 0.137; *p* = 0.006), and the FSU (β = 0.270; *p* = 0.004). The direct effect of benefit on contact was also positive for asylum seekers, but its size did not reach the required level of significance (β = 0.113; *p* = 0.03). Thus, the results obtained mostly confirmed the hypothesis regarding the effect of benefit on contact and mostly did not confirm the hypothesis regarding the effect of threat on contact across different immigrant groups.

Among all direct effects of values on contact, only the effect of conservation on contact was significant for two diaspora immigrant groups: immigrants from western countries and immigrants from the FSU. In addition, some indirect effects of values on contact were significant in different groups: conservation for asylum seekers (β = −0.046; *p* = 0.008), self-transcendence for immigrants from Ethiopia (β = 0.098; *p* = 0.001), self-enhancement for immigrants from Ethiopia (β = −0.075; *p* = 0.007) and the FSU (β = −0.069; *p* = 0.003), and openness to change for immigrants from western countries (β = 0.040; *p* = 0.003). Thus, although the size of effects varied, growth anxiety-free values were positively related to contact, while anxiety-avoidance values were negatively related to contact across different immigrant groups.

### Direct Effects of Personal Values on Threat-Benefit Appraisal

Examining the direct effects of values on benefit, we found that the effect of openness to change on benefit was positive regarding all groups of immigrants, but in no group it reached the required level of significance. The effect of self-transcendence on benefit was positive and significant regarding asylum seekers and immigrants from Ethiopia. The effect of conservation on benefit was negative and significant regarding asylum seekers. The effect of self-enhancement on benefit was negative and significant regarding immigrants from Ethiopia.

Examining the direct effects of values on threat, we found that the effect of openness to change on threat was negative and significant regarding immigrants from Ethiopia and western countries. The effect of self-transcendence was negative and significant regarding asylum seekers. The effect of conservation on threat in no group reached the required level of significance. Finally, the effect of self-enhancement on threat was positive and significant among all four groups. In general, the pattern of connections between values and appraisal of immigrants confirmed the hypothesis across the four immigrant groups.

The results obtained demonstrated that direct connections between the number of immigrants in one’s social surrounding and contact with them were positive and significant for both the number of immigrants in the residential area and at work regarding all immigrant groups: asylum seekers (β = 0.140; *p* = 0.002; β = 0.518; *p* = 0.002), immigrants from Ethiopia (β = 0.257; *p* = 0.002; β = 0.344; *p* = 0.001), western countries (β = 0.271; *p* = 0.002; β = 0.358; *p* = 0.002), and the FSU (β = 0.217; *p* = 0.001; β = 0.290; *p* = 0.002). Numbers of immigrants in the place of work and in the residential area were not related to their appraisal for all immigrant groups, except asylum seekers, for whom their number at work was positively and significantly related to their appraisal as beneficial (β = 0.223; *p* = 0.001).

### Indirect Effects Between Personal Values and Contact

Significant positive indirect effects through both threat and benefit were found for openness to change and contact for Ethiopian and Western targets. Significant indirect effects (through threat only) were also found for the value of self-transcendence for asylum seeker targets and (through benefit only) for Ethiopian and FSU targets. A significant indirect relationship between conservation and contact was found only for asylum seekers (through both threat and benefit), while a significant negative indirect relationship between self-enhancement and contact was found for Ethiopian (through both threat and benefit) and FSU immigrants (through threat). See Figure 3 for significant direct effects for each of the four groups and the online supplement for tables presenting all direct and indirect effects separately for the four groups.

## Discussion

The current study tested a theoretical model examining the relationships between personal value preferences (Schwartz et al., 2012) and appraisal of immigrant groups as representing both a threat and a benefit to the receiving population and their association with levels of social contact. The goodness of fit measures for the theoretical model and relatively high levels of explained variance confirmed hypotheses that personal values predicted levels of appraisal, which in turn, together with opportunities for contact, predicted levels of social contact. Findings support a theoretical model in which personal values predict social contact both directly (in the case of conservation values) but also, and more notably, indirectly, through their impact on positive and negative appraisal of the immigrant group. They also extend previous research on personal values and attitudes to immigration (Davidov et al., 2008; Davidov and Meuleman, 2012; Beierlein et al., 2016; Davidov et al., 2020) by showing how they predict levels of social contact.

Higher levels of anxiety avoidance values (self-enhancement and conservation) and lower levels of anxiety-free values (openness to change and self-transcendence) predicted higher levels of perceived threat while lower levels of conservation values and higher levels of self-transcendence values predicted higher levels of perceived benefit. Results suggest that individuals, who are motivated by higher levels of anxiety, as manifested in values of conservation and self-enhancement, are more likely to perceive immigrant members as a threat and resist social contact. These results support previous findings (Davidov et al., 2020) and strengthen a synthesis between ITT (Stephan and Stephan, 1996; Stephan and Stephan, 2000) and values theory (Schwartz, 2006) in which higher levels of anxiety lead to greater perceptions of threat. The positive relationship between self-transcendence and benefits may be explained through previous research showing the relationship between self-transcendence values and the ability to be empathic and sensitive to others (Caprara et al., 2012; Zibenberg and Kupermintz, 2016). We can suggest that the ability to take another’s perspective can enable the appreciation of the benefits they bring with them. Similarly, the need for individuals high on conservation values to preserve the status quo (Schwartz et al., 2012) and the anxiety they may feel when confronted with new possibilities, would seem to make it difficult for them to appreciate the benefits that a new group can bring. Findings also strengthen the idea that appraisal of the other and establishing contact with him/her can be a means through which the individual can attain or fulfill general motivational goals expressed in personal value preferences (Sagiv et al., 2004).

Results emphasize the importance of a model of intergroup relations that takes into account both the threats and the benefits that an immigrant group may represent to the local population. Previous models have tended to emphasize the role of symbolic and realistic threat (Stephan and Stephan, 2000; Stephan et al., 2005) in predicting attitudes toward immigrants, but the current findings suggest that a more holistic perspective, in which immigrant groups may represent a host of possible benefits (economic, cultural diversity, humanitarian and social cohesion) for the receiving population is imperative for understanding what can enhance inter-group relationships (Thravalou et al., 2020). As such our model, advancing previous studies (Raijman et al., 2008), supports a two-factor model of appraisal. It is also important, both theoretically and practically to acknowledge that positive, and not just negative appraisal impacts on levels of social contact. Indeed, positive appraisal was a better predictor of contacts than negative appraisal. For only one of the groups (FSU immigrants) was there a significant direct association between threats and contact such that a perspective focusing on negative appraisal alone would not only fail to show the indirect relationships between values and contact but would also give a partial conceptual understanding of the relationship between appraisal and contact. On a theoretical level, the model reinforces a values-attitude-behavior paradigm (Homer and Kahle, 1988). From this perspective, general motivational goals affect how a person forms his/her specific attitudes, which in turn affect his/her behavior toward the specific object when a social situation provides an opportunity for the desired behavior. The direct effect of values on behavior is weak because values are too abstract as concepts. However, the conservation values may be directly related to avoiding immigrants, because they are more strongly associated with anxiety than other values (Nelissen et al., 2007; Tamir et al., 2016).

Levels of opportunities for contact, both in the neighborhood and the workplace were not directly related to levels of appraisal, suggesting that levels of appraisal may be related to more personality (values-) based than situationally based anxiety (Bouchard et al., 2004; Averill, 2015). While opportunities for contact may not change attitudes toward the group as a whole, they enable the local population to meet, interact, and potentially like individuals in the immigrant community (who they may work with or live near). Such social contact may even be despite their general appraisal of the group to which the immigrants belong.

While the general model held across groups, there were some differences in the relationships between values and appraisal among the four groups. Higher levels of benefits were associated with higher levels of social contact for all four groups, yet appraisal of an immigrant group as a threat was associated with less social contact only for immigrants from the FSU. While further research is needed to understand why this would be, we can suggest that it may be related to the number of immigrants and/or their social power and capital (Amit, 2012). In line with SCM (Fiske et al., 2007; Cuddy et al., 2008, 2009), FSU immigrants represent low warmth (i.e., high competition), and relatively high competence (high social status) which may make their threat more actual. The perceived threats for the society may prevent locals from establishing personal contact. It is interesting to examine the group differences through a SCM lens. For example, the positive relationship between self-transcendence and benefits for asylum seekers and Ethiopian immigrants and the negative relationship between self-transcendence and threat for asylum seekers may be explained by low levels of perceived competence for both groups (i.e., low social status will incline individuals for whom universalism and benevolence are important to feel greater positive and less negative appraisal). Similarly, a negative relationship between conservation and benefit for asylum seekers may be explained by their perceived status as low warmth (i.e., high competition). Overall, it is interesting that there were more associations between values and appraisal for the asylum seekers and the Ethiopian immigrants than for the FSU and Western immigrants. Connections between values and appraisal were also, in general, stronger for asylum seekers and Ethiopians than for FSU and Western immigrants. In line with SCM, we hypothesize that in cases where immigrant groups are larger and opportunities for contact are greater, the role of internal personality factors may be lower (Grigoryev et al., 2019b). Real contact, in such cases, may reduce the role of individual factors. The smaller the group, the lesser the contact and the less the individual has the chance to experience members of the group, the more values and stereotypes may predict contact.

It is also interesting to understand the differences between the groups in the indirect relationships between values and contact within an SCM lens. For example, positive indirect effects were found between openness to change and contact, through higher levels of benefits and lower levels of threats, for both Ethiopian and Western immigrants (both considered high on warmth (i.e., low competition). It may be that in the context of low competition, individuals with high levels of openness to change will be more confident to appraise incoming groups positively, thus feeling more secure in pursuing contact. As might be predicted, the negative indirect relationship between conservation and contact (through higher levels of the perceived threat and lower levels of perceived benefits) was found only for asylum seekers who represent high competition (a high threat to the status quo). Results suggest that the SCM is a helpful framework through which to understand a threats-benefits profile of specific immigrant group (“mild” outgroups, “moderate” outgroups, and “extreme” outgroups) as it provides a more nuanced understanding of how immigrant groups are perceived by the host society (Dricu et al., 2020).

### Limitations

The current study involved a large representative sample of adults within the majority Jewish population in Israel and examined four diverse groups of immigrants. Despite this, further research would be needed to examine the validity of the model in additional cultural contexts and with further immigrant groups. Israel is a country that encourages cultural assimilation of diaspora immigrants (Horenczyk and Ben-Shalom, 2006), and it would be important to examine the model in countries that encourage greater levels of cultural diversity patterns (Bourhis et al., 1997). In addition, we decided to examine the majority population to test the conceptual model. It would be important to see whether the study model is applicable for members of minority populations in a society (e.g., Palestinian Israelis) and between immigrant groups (e.g., appraisal of immigrants from the FSU by Ethiopian immigrants). The cross-sectional nature of the study does not allow the assertion of causality, nor does it allow a complete assessment of mediation (Kline, 2015; O’Laughlin et al., 2018) although the model rests on theoretical constructs (Hill, 1965). Therefore, further experimental and longitudinal studies are needed to test the model and to ascertain mediation and causality. In addition, while the current study examines direct and indirect relationships between values and contact, recent literature emphasizes the interaction between personality variables and context (Cohrs and Asbrock, 2009; Araújo et al., 2020). Future research should examine the moderation effects of contextual variables on the presented model. Finally, the differences in sample sizes for the pooled sample vs. the individual groups, means that there was a difference in statistical power between these analyses.

## Implications and Conclusion

The conceptual model examined in the current study reveals the effects of personal variables on social contact with immigrants among the local population, and, as such, it has several important theoretical contributions. It expands traditional theories of threat (Stephan and Stephan, 2000) to stress the importance of a more comprehensive model of appraisal which includes both negative and positive aspects of perception of the “other.” It also suggests that the individual’s appraisal of an immigrant group as representing various threats and/or benefits to the society is associated with the personal value preferences that the individual holds and that this appraisal is associated with the behavioral choices the individual makes around social contact with members of the immigrant group. In addition, in line with SCM, results suggest that a comprehensive model examining values-appraisal-behavior should include a nuanced view taking into account the particular social structural characteristics of the immigrant group. Further study could examine additional behaviors that may be predicted by a values-appraisal perspective such as affirmative action, granting minority rights or permanent status, or support for pro-minority policies.

On a practical level, a means of understanding what can predict or possibly enable more social contact can be important for promoting more positive intergroup relationships in heterogeneous societies (Van Laar et al., 2005). The model suggests that to enhance intergroup relations more emphasis can be put on values-based interventions and education (Biesta, 2010; Bardi and Goodwin, 2011); e.g., strengthening values of self-transcendence and openness to change, and policy emphasizing the contributions of immigrant groups to the local society (Florack et al., 2003). However, results also suggest that values-based messaging should be nuanced according to the characteristics of the particular immigrant group and their position in the host society. For example, study results suggest that while self-transcendence-based messaging on benefits can persuade people to be in contact with immigrants from low-status groups, it is unlikely to be useful for promoting contact with high and medium-level status groups. Results also challenge immigration policies that segregate immigrants into highly concentrated immigrated neighborhoods (Hall, 2013) and do not encourage the integration of immigrants in the workplace and social environments. Such policies prevent locals from establishing important and satisfying social contacts with immigrants.

## Data Availability Statement

The raw data supporting the conclusions of this article will be made available by the authors, without undue reservation.

## Ethics Statement

The studies involving human participants were reviewed and approved by the Bar-Ilan University Ethical Board. The patients/participants provided their written informed consent to participate in this study.

## Author Contributions

SW led the writing of the manuscript. ET was involved in the writing and editing and approved the final draft. Both authors equally contributed in the conceptualization of the research project, the designing and overseeing of the research project and the analysis.

## Conflict of Interest

The authors declare that the research was conducted in the absence of any commercial or financial relationships that could be construed as a potential conflict of interest.

## Footnotes

1 http://www.cbs.gov.il/shnaton68/st02_08x.pdf

2 https://www.cbs.gov.il/en/subjects/Pages/Immigration-and-International-Migration.aspx

3 Israel is composed of a majority (Jewish) and a minority (Arab) population. In the current study, we examine the theoretical model to assess and understand the appraisal of immigrants by the majority population. We expect that threat/benefit appraisal would differ between members of the majority and minority population due to their different levels of power and influence, and this will need to be examined in follow-up research.

4 https://directory.esomar.org/country87_Israel/r487_PORI.php

5 For all questionnaires, the word immigrant was substituted with one of the following: immigrants from the FSU, immigrants from Ethiopia, immigrants from western countries such as France, the United Kingdom, or the United States, asylum seekers.

6 To avoid cluttering, the effects of socio-demographic variables are not presented in the path diagram; however, they are presented in Supplementary Figure A1. Correlations between disturbances of the threat and benefit, as well as between the numbers of immigrants at one’s place of work and in the residential area are presented in the path diagram. However, to avoid clattering, disturbances of other variables and correlations between them, as well as correlations between exogenous variables, are not presented in the path diagram. They are available from the authors upon request.
