# Supplementary figures and images for: Personal Value Preferences, Threat-Benefit Appraisal of Immigrants and Levels of Social Contact: Looking Through the Lens of the Stereotype Content Model
Source: Front Psychol. 2021 Mar 4;12:609219. doi: 10.3389/fpsyg.2021.609219 (PMC7970186; doi:10.3389/fpsyg.2021.609219)

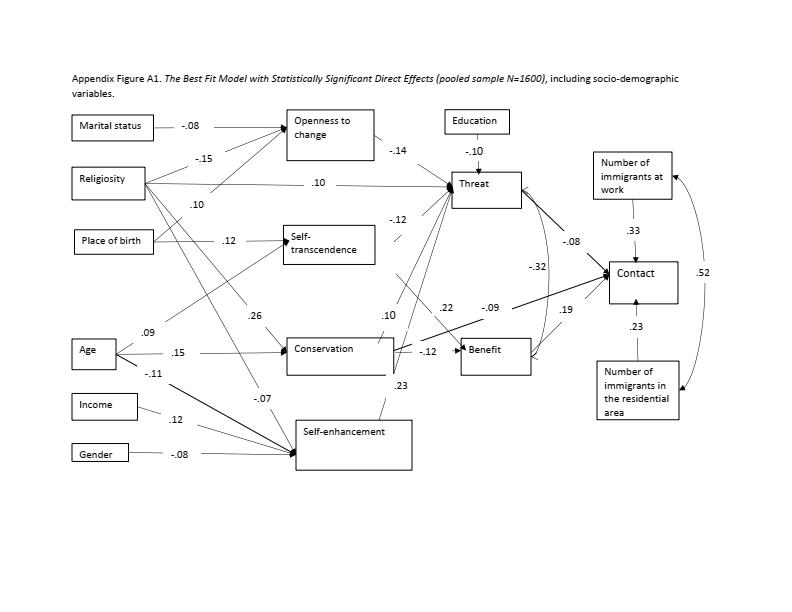

Supplement: Supplementary file 1 [file Image_1.jpg]
