# Supplementary material for: Personal Value Preferences, Threat-Benefit Appraisal of Immigrants and Levels of Social Contact: Looking Through the Lens of the Stereotype Content Model
Source: Front Psychol. 2021 Mar 4;12:609219. doi: 10.3389/fpsyg.2021.609219 (PMC7970186; doi:10.3389/fpsyg.2021.609219)
Supplement: Supplementary file 2 [file Table_1.DOCX]

**Online supplement: A1. Standardized Direct, Indirect and Total Effects for the Multi-group Analysis**

Table 1. *Multi-Group Model. Standardized Direct Effects: Estimate, Standard Error, and Level of Significance*

| **Var.** | **Asylum Seekers** | |  | **Ethiopians** |  |  | **Western Countries** | |  | **Former Soviet Union** | | |
| --- | --- | --- | --- | --- | --- | --- | --- | --- | --- | --- | --- | --- |
|  | **Benefit** | **Threat** | **Contact** | **Benefit** | **Threat** | **Contact** | **Benefit** | **Threat** | **Contact** | **Benefit** | **Threat** | **Contact** |
| Threat |  |  | -.04(.05) |  |  | -.04(.05) |  |  | -.07(.05) |  |  | -.18(.05)^**^ |
| Benefit |  |  | .11(.05)^*^ |  |  | .26(.05)^**^ |  |  | .14(.05)^**^ |  |  | .27(.04)^**^ |
| O2CH | .16(.07)^*^ | -.01(.07) | -.02(.06) | .20(.07)^*^ | -.21(.07)^* *^ | .03(.07) | .14(.07)^*^ | -.27(.07)^**^ | .10(.06) | .06(.07) | -.12(.07) | .10(.06) |
| SETR | .51(.08)^**^ | -.31(.08)^**^ | .08(.07) | .36(.07)^* *^ | -.14(.08) | -.03(.07) | .11(.08) | -.03(.08) | .11(.07) | .18(.08)^*^ | -.11(.08) | -.02(.07) |
| CONS | -.34(.07)^**^ | .18(.07)^*^ | -.04(.0) | -.11(.07)^*^ | -.06(.07) | .01(.06) | .00(.08) | .18(.08)^*^ | -.20(.07)^**^ | -.06(.07) | .14(.07) | -.16(.06)^**^ |
| SENH | .11(.06) | .15(.06)^**^ | .00(.05) | -.22(.06)^**^ | .48(.06)^**^ | -.05(.06) | .11(.06) | .16(.06)^**^ | -.09(.05) | -.05(.06) | .27(.06)^**^ | .06(.05) |
| N 2 | .09(.05) | .02(.05) | .14(.04)^**^ | -.05(.05) | -.01(.05) | .26(.04)^**^ | -.08(.06) | -.03(.05) | .27(.05)^**^ | .07(.05) | -.04(.05) | .22(.04)^***^ |
| N 1 | .22(.05)^**^ | -.08(.05) | .52(.04)^**^ | .04(.05) | .03(.05) | .34(.05)^** *^ | .04(.06) | .03(.05) | .36(.05)^**^ | .08(.05) | .10(.05) | .29(.05)^**^ |
| Gender | .09(.05) | -.06(.05) | -.03(.04) | .07(.05) | -.07(.05) | .04(.04) | .03(.05) | -.03(.05) | -.01(.04) | -.02(.05) | -.02(.05) | .06(.04) |
| Age | -.01(.05) | .03(.05) | -.02(.04) | .09(.05) | -.04(.05) | -.10(.04)^*^ | .12(.06) | .01(.05) | -.07(.04) | .09(.05) | .03(.05) | -.11(.04)^*^ |
| Ed. | -.07(.05) | -.08(.05) | .01(.04) | .01(.05) | -.13(.04)^*^ | -.04(.04) | .08(.05) | -.10(.05)^*^ | .10(.04) | .12(.05)^*^ | -.08(.05) | .03(.04) |
| Rel. | -.11(.06)^*^ | .18(.06)^**^ | -.07(.05) | .12(.06)^*^ | .05(.06) | .02(.05) | .01(.06) | .09(.06) | .04(.05) | -.09(.06) | .15(.06)^*^ | .07(.06) |
| Income | .10(.05) | .06(.05) | .04(.05) | .10(.05) | .08(.05) | .09(.05) | .00(.05) | .03(.05) | -.04(.06) | .05(.05) | -.03(.05) | .09(.05) |
| Marital status | .04(.05) | .12(.05)^*^ | -.06(.05) | -.07(.05) | -.04(.05) | -.11(.05)^*^ | .01(.05) | -.06(.05) | -.09(.05) | -.10(.05) | .01(.05) | -.02(.05) |
| Place of birth | .02(.05) | -.01(.05) | .09(.04)^*^ | .13(.05)^*^ | -18(.05)^***^ | .02(.05) | -.01(.05) | .01(.05) | -.01(.05) | -.05(.05) | -.03(.05) | .02(.05) |

*Note*: ^*^ *p* < .05; ^**^ *p* < .01; ^***^ *p* < .001. Var. – predicting variables; Ed. – the level of education; Rel. – the level of religiosity; N 1 – number of immigrants at one’s work; N 2 – number of immigrants in the residential area; CONS – Conservation; SETR – Self-transcendence; SENH – Self-enhancement; O2CH – Openness to change. Gender: 1 – male; 2 – female. Marital status: 0 – single; 1 – living with a partner. Place of birth: 0 – foreign born; 1 – born in Israel.

Table A2. *Multi-Group Model. Standardized Indirect Effects: Estimate, Standard Error, and Level of Significance*

| **Var.** | **Asylum Seekers** | | |  | **Ethiopians** |  |  | **Western Countries** | |  | **Former Soviet Union** | | |
| --- | --- | --- | --- | --- | --- | --- | --- | --- | --- | --- | --- | --- | --- |
|  | **Benefit** | | **Threat** | **Contact** | **Benefit** | **Threat** | **Contact** | **Benefit** | **Threat** | **Contact** | **Benefit** | **Threat** | **Contact** |
| O2CH |  | |  | -.02(.01) |  |  | .06(.03)^*^ |  |  | .04(.02)^**^ |  |  | .04(.03) |
| SETR |  | |  | .07(.03)^*^ |  |  | .10(.03)^**^ |  |  | .02(.02) |  |  | .06(.03)^*^ |
| CONS |  | |  | -.05(.02)^**^ |  |  | -.03(.03) |  |  | -.01(.02) |  |  | -.04(.03) |
| SENH |  | |  | .01(.01) |  |  | -.08(.03)^**^ |  |  | .01(.02) |  |  | -.07(.02)^**^ |
| N 2 |  | |  | .01(.01) |  |  | .01(.02) |  |  | .01(.01) |  |  | .03(.02) |
| N 1 |  | |  | .03(.01)^**^ |  |  | .01(.02) |  |  | -.01(.01) |  |  | .01(.02) |
| Gender | .01(.02) | | -.03(.02)^*^ | .02(.01) | -.02(.02) | .02(.02) | .02(.02) | .01(.02) | .01(.02) | .00(.02) | .02(.01) | -.02(.02) | -.01(.02) |
| Age | .01(.02) | | -.03(.02) | .01(.01) | -.01(.03) | -.03(.03) | .03(.02) | .01(.02) | .01(.02) | -.01(.02) | -.01(.02) | .01(.02) | -.04(.02) |
| Ed. | .01(.02) | | .01(.02) | .00(.01) | .02(.02) | -.03(.02) | .02(.02) | .01(.02) | -.03(.01)^*^ | .04(.01) ^**^ | .02(.01) | .01(.02) | .06(.02)^**^ |
| Rel. | -.10(.03)^***^ | | .05(.03) | -.05(.02)^*^ | -.06(.02)^*^ | -.01(.02) | .02(.02) | -.04(.03) | .07(.03)^*^ | -.08(.03)^**^ | -.04(.03) | .07(.03)^*^ | -.13(.03)^**^ |
| Income | | -.03(.02) | .02(.02) | -.01(.01) | -.01(.02) | .05(.02) | .01(.02) | .02(.02) | .04(.02)^*^ | -.03(.02) ^*^ | .01(.02) | .02(.02) | -.02(.02) |
| Marital status | | .01(.02) | .00(.02) | .01(.01) | -.02(.02) | -.01(.02) | -.02(.02) | -.01(.02) | .03(.02) | -.01(.02) | -.02(.02) | .03(.02) | .01(.02) |
| Place of birth | | .02(.02) | -.04(.02)^*^ | .01(.01)^*^ | .05(.02) ^*^ | .01(.02) | .05(.02)^*^ | .03(.02) | -.01(.02) | .01(.02) | .02(.02) | .01(.02) | .01(.02) |

*Note*: ^*^ *p* < .05; ^**^ *p* < .01; ^***^ *p* < .001. Var. – predicting variables; Ed. – the level of education; Rel. – the level of religiosity; N 1 – number of immigrants at one’s work; N 2 – number of immigrants in the residential area; CONS – Conservation; SETR – self-transcendence; SENH – self-enhancement; O2CH – openness to change. Gender: 1 – male; 2 – female. Marital status: 0 – single; 1 – living with a partner. Place of birth: 0 – foreign born; 1 – born in Israel.

Table A3. *Multi-Group Model. Standardized Total Effects: Estimate, Standard Error, and Level of Significance*

| **Var.** | **Asylum Seekers** | |  | **Ethiopians** |  |  | **Western Countries** | |  | **Former Soviet Union** | | | | |  |
| --- | --- | --- | --- | --- | --- | --- | --- | --- | --- | --- | --- | --- | --- | --- | --- |
|  | **Benefit** | **Threat** | **Contact** | **Benefit** | **Threat** | **Contact** | **Benefit** | **Threat** | **Contact** | **Benefit** | **Threat** | | | **Contact** |  |
| Threat |  |  | -.04(.04) |  |  | -.04(.05) |  |  | -.08(.05) |  | |  | -.18(.05)^**^ | | |
| Benefit |  |  | .11(.05)^*^ |  |  | .26(.05)^**^ |  |  | .14(.05)^**^ |  | |  | .27(.04)^**^ | | |
| CONS | -.34(.07)^***^ | .18(.07)^*^ | -.09(.06) | -.11(.07) | -.06(.06) | -.02(.06) | .01(.08) | .18(.08)^*^ | -.22(.07)^**^ | -.07(.07) | | .14(.07) | -.21(.06)^**^ | | |
| SETR | .51(.08)^***^ | -.31(.08)^***^ | .15(.07) | .36(.07) ^**^ | -.14(.08) | .07(.07) | .11(.08) | -.03(.08) | .13(.07) | .18(.08)^*^ | | -.11(.08) | .05(.07) | | |
| SENH | .11(.06) | .15(.06)^**^ | .01(.05) | -.22(.06)^**^ | .48(.06)^**^ | -.13(.05)^*^ | .11(.06) | .16(.06)^**^ | -.08(.05) | -.05(.06) | | .26(.06)^**^ | .01(.05) | | |
| O2CH | .16(.07)^*^ | -.01(.07) | -.04(.06) | .20(.07)^*^ | -.21(.07)^* **^ | .09(.07) | .14(.07)^*^ | -.27(.07)^**^ | .12(.06)^*^ | .07(.07) | | -.11(.07) | .12(.07) | | |
| N 2 | .09(.05) | .02(.05) | .15(.04)^**^ | -.06(.05) | -.01(.05) | .25(.05)^**^ | -.08(.06) | -.04(.05) | .26(.05)^**^ | .07(.05) | | -.04(.05) | .24(.04)^**^ | | |
| N 1 | .22(.05)^***^ | -.08(.05) | .55(.04)^***^ | .04(.05) | .03(.05) | .35(.04)^** *^ | .04(.06) | .03(.05) | .36(.05)^**^ | .08(.05) | | .09(.05) | .31(.05)^**^ | | |
| Gender | .10(.05)^*^ | -.08(.05) | -.01(.04) | .06(.05) | -.04(.05) | .05(.04) | .03(.05) | -.02(.05) | -.01(.04) | -.01(.05) | | .04(.05) | .03(.04) | | |
| Age | .01(.05) | -.06(.05) | -.01(.04) | .08(.05) | -.06(.05) | -.07(.04)^*^ | .13(.05) | .04(.05) | -.04(.04) | .08(.05) | | .04(.05) | -.15(.04)^**^ | | |
| Ed. | -.06(.05) | -.08(.05) | .01(.04) | .02(.05) | -.16(.04) ^**^ | -.02(.04) | .07(.05) | -.12(.05)^*^ | .15(.04) ^*^ | .14(.05)^**^ | | -.08(.05) | .11(.04)^**^ | | |
| Rel. | -.21(.06)^**^ | .23(.06)^**^ | -.11(.05)^**^ | .06(.06) | .04(.06) | .04(.05) | -.04(.06) | .17(.06)^**^ | -.04(.05) | -.15(.06)^**^ | | .23(.06)^**^ | -.07(.06) | | |
| Income | .07(.05) | .08(.05) | .04(.05) | .09(.06) | .13(.06)^*^ | .10(.06) | .02(.05) | .07(.05) | -.07(.06) | .05(.05) | | -.02(.05) | .07(.05) | | |
| Marital status | .05(.05) | .12(.05)^*^ | -.06(.05) | -.09(.05) | -.05(.06) | -.13(.06) ^*^ | -.01(.05) | -.03(.05) | -.10(.05) | -.12(.05) | | .01(.05) | -.01(.05) | | |
| Place of birth | .05(.05) | -.05(.05) | .10(.04)^*^ | .19(.05)^**^ | -.17(.05) ^**^ | .07(.05) | .02(.05) | .00(.05) | -.01(.05) | -.06(.05) | | -.03(.05) | .03(.05) | | |

*Note*: ^*^ *p* < .05; ^**^ *p* < .01; ^***^ *p* < .001. Var. – predicting variables; Ed. – the level of education; Rel. – the level of religiosity; N 1 – number of immigrants at one’s work; N 2 – number of immigrants in the residential area; CONS – Conservation; SETR – self-transcendence; SENH – self-enhancement; O2CH – openness to change. Gender: 1 – male; 2 – female. Marital status: 0 – single; 1 – living with a partner. Place of birth: 0 – foreign born; 1 – born in Israel.

**2. Socio-demographic Variables, Appraisal and Social Contact**

In the pooled sample, seven socio-demographic variables were included as controls. As the results demonstrated, no direct effect of socio-demographic variables on contact was significant. However, two indirect effects of two socio-demographic variables on contact were significant: education (*β* = .016; *p* = .005) and religiosity (*β* = -.052; *p* = .002). All direct effects of socio-demographic variables on benefit were not significant. However, direct effects of two socio-demographic variables on threat were significant: education (*β* = -.100; *p* = .001) and religiosity (*β* = .102; *p* = .002). Moreover, indirect effects of the following socio-demographic variables on threat were significant: religiosity (*β* = .038; *p* = .002), income (*β* = .023; *p* = .001), and place of birth (*β* = -.019; *p* = .001). Finally, indirect effects of the following socio-demographic variables on benefit were significant: religiosity (*β* = -.051; *p* = .002) and place of birth (*β* = .023; *p* = .001). The indirect effects of socio-demographic variables on threat and benefit were due to their connections with values: income was connected to self-enhancement (*β* = .117; *p* = .002); place of birth was connected to openness to change (*β* = .099; *p* = .002) and self-transcendence (*β* = .116; *p* = .001); and religiosity was connected to openness to change (*β* = -.150; *p* = .002), conservation (*β* = .257; *p* = .001), and self-enhancement (*β* = -.073; *p* = .004).

In the four separate group models gender, age, education, income, and marital status were not significantly related to either appraisal or contact with immigrants. However, the level of religiosity was positive and significantly related to threat regarding asylum seekers (*β* = .181; *p* = .003), and the place of birth was significantly related to threat regarding immigrants from Ethiopia, such that Israelis born abroad appraised immigrants from Ethiopia as a greater threat than people born in Israel (*β* = -.179; *p* = .005).

While effects of socio-demographic variables were relatively weak, there were some significant relationships which both strengthen extensive previous literature on the relationships between gender, age, education and religion on personal values and appraisal (Schwartz and Rubel, 2005;Schwartz, 2006;Hainmueller and Hiscox, 2007;Vecchione et al., 2012), and also point out distinctive characteristics of Israel and the particular immigrant groups. Notably, overall, religiosity was the most influential of the socio-demographic variables which may be related to the centrality of Judaism in local members identity and the relationship between Jewish status and immigration laws in Israel (Bourhis and Dayan, 2004). It is also interesting that there were no direct relationships between socio-economic variables and social contact in the general model strengthening understandings that their impact on behavior is through psychological mechanisms (in this case mainly through the relationship with values).
